# Vasoinhibin comprises a three-helix bundle and its antiangiogenic domain is located within the first 79 residues

**DOI:** 10.1038/s41598-018-35383-7

**Published:** 2018-11-20

**Authors:** Juan Pablo Robles, Magdalena Zamora, José Luis Velasco-Bolom, Miriam Tovar, Ramón Garduño-Juárez, Thomas Bertsch, Gonzalo Martínez de la Escalera, Jakob Triebel, Carmen Clapp

**Affiliations:** 1 Instituto de Neurobiología, Universidad Nacional Autónoma de México (UNAM), Querétaro, Mexico; 2 Biofísica y Ciencia de Materiales, Instituto de Ciencias Físicas, UNAM, Cuernavaca, Mexico; 3 Institute for Clinical Chemistry, Laboratory Medicine and Transfusion Medicine, Nuremberg General Hospital & Paracelsus Medical University, Nuremberg, Germany

## Abstract

Vasoinhibin belongs to a family of angiogenesis inhibitors generated when the fourth α-helix (H4) of the hormone prolactin (PRL) is removed by specific proteolytic cleavage. The antiangiogenic properties are absent in uncleaved PRL, indicating that conformational changes create a new bioactive domain. However, the solution structure of vasoinhibin and the location of its bioactive domain are unknown. Molecular dynamic simulation (MD) showed that the loss of H4 exposes the hydrophobic nucleus of PRL and leads to the compression of the molecule into a three-helix bundle that buries the hydrophobic nucleus again. Compression occurs by the movement of loop 1 (L1) and its interaction with α-helix 1 (H1) generating a new L1 conformation with electrostatic and hydrophobic surfaces distinct from those of PRL, that may correspond to a bioactive domain. Consistent with this model, a recombinant protein containing the first 79 amino acids comprising H1 and L1 of human PRL inhibited the proliferation and migration of endothelial cells and upregulated the vasoinhibin target genes, IL1A and ICAM1. This bioactivity was comparable to that of a conventional vasoinhibin having the 123 residues encompassing H1, L1, Η2, L2, and Η3 of human PRL. These findings extend the vasoinhibin family to smaller proteins and provide important structural information, which will aid in antiangiogenic drug development.

## Introduction

Angiogenesis, the formation of new blood vessels from pre-existing vasculature, regulates the growth of tissues during development and in adult life, and abnormal angiogenesis underlies the progression of multiple diseases including cancer, arthritis, diabetic retinopathy, neurodegeneration, pre-eclampsia, and peripartum cardiomyopathy^1,2^. Therefore, drugs that target the growing vasculature are promising therapeutics^3^. Many inhibitors of angiogenesis are cleaved derivatives of endogenous proteins with no antiangiogenic activity, including extracellular matrix and basement membrane proteins, growth factors, cytokines, circulating proteins, and hormones^4,5^.

Prolactin (PRL), the pituitary hormone essential for lactation, is proteolytically converted to vasoinhibin, which belongs to a protein family, including 16 K PRL, that inhibits the proliferation, survival, permeability, and dilation of blood vessels^6,7^. Inhibition of endothelial cell proliferation, migration, and survival involve the binding of vasoinhibin to a multicomponent complex conformed by plasminogen activator inhibitor-1, urokinase plasminogen activator, and the urokinase plasminogen activator receptor on endothelial cell membranes^8^. These, but also other unidentified binding partners/receptors^7,9^, mediate vasoinhibin inhibition of the signalling pathways (Ras-Raf-MAPK; Ras-Tiam1-Rac1-Pak1; PI3K-Akt and PLCγ-IP3-eNOS) activated by several proangiogenic factors (VEGF, bFGF, bradykinin, IL-1β)^6–8^. The generation of vasoinhibin is regulated at the hypothalamus, the pituitary, and the target tissue levels defining the PRL/vasoinhibin axis^7^. Disruption of this axis contributes to the pathogenesis and progression of diabetic retinopathy^10^, retinopathy of prematurity^11^, peripartum cardiomyopathy^12^, and pre-eclampsia^13^.

**Figure 1:**
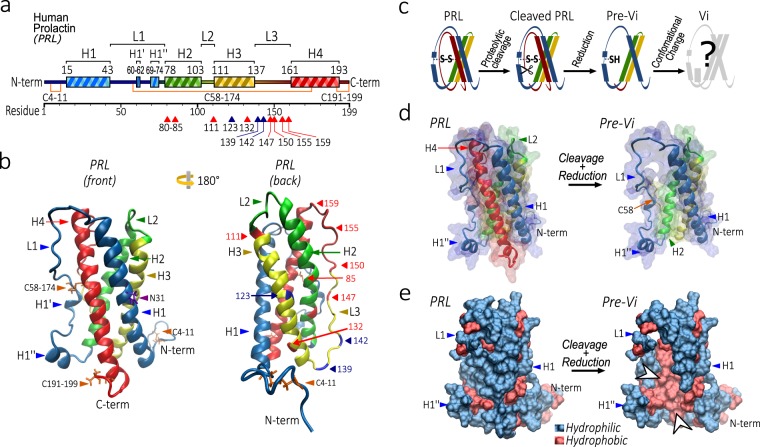
Structural description of prolactin (PRL). (**a**) Linear representation of human PRL secondary structure. The number of amino acids comprising the *α*-helix structures (H1-4), the loops (L1-3), and the three disulphide bonds are indicated. The vasoinhibin (Vi) generated by protease cleavage at different sites is indicated by red arrows and corresponding cleavage residue numbers. Vi produced by recombinant technology are indicated by blue arrows and the number of the terminal residue. (**b**) 3D Ribbon representation of soluble human PRL (PDB entry 1RW5). After a 180° turn of the structure, sites of protease cleavage in L3, in H3, and in H2 are indicated (red arrows and numbers). Terminal residues of recombinant vasoinhibins are also indicated (blue arrows and numbers). (**c**) Steps required for the generation of vasoinhibin from PRL. (**d**) The PRL and a pre-vasoinhibin (Pre-Vi) of 150 residues are shown in a ribbon representation superimposed on a translucent surface model. (**e**) Surface representation of hydrophilic (blue) and hydrophobic (red) residues in PRL and Vi showing the hydrophobic nuclei (white arrows) exposed after removal of H4.

The fact that full-length PRL is devoid of antiangiogenic properties indicates that an antiangiogenic domain encrypted within PRL becomes exposed by proteolytic cleavage. The functional consequences of such processing highlight the need to understand the structural transition of PRL to vasoinhibin and the location of the newly exposed biological determinant. The solution structure of vasoinhibin is unknown, but that of PRL has been defined^14^. Human PRL comprises 199 residues that adopt an “up-up-down-down” four α-helical (H1-4) bundle topology connected by three loops (L1-3) and flanked by small N-terminal and C-terminal loops (Fig. 1a,b). L1 contains two small helices, 3_10_-helix (H1’) and α-helix (H1”), and the whole molecule is stabilized by three disulphide bonds forming two small loops (C4-11 and C191-199) in the terminal regions and a long loop (C58-174) that links L1 with H4 (Fig. 1a,b). Cathepsin D^15^, matrix metalloproteinases (MMP)^16^, and bone morphogenetic protein-1^17^ cleave at various sites of human PRL to generate peptides that range from the first 80 to the first 159 residues (Fig. 1a,b). PRL fragments having 123 or more amino acids have been tested for vasoinhibin activity^6,7^. The bioactive fragments have in common the loss of the H4 and the C-terminal loop due to both the proteolytic cleavage and the reduction of the C58-174 disulphide bond (Fig. 1c)^6,7^. This observation implies that the loss of these regions creates a new bioactive conformation.

Here, we used molecular dynamic (MD) simulation to study the structural changes induced by the loss of H4 in human PRL and the putative location of the vasoinhibin bioactive domain. The work revealed that after removal of H4, PRL compresses into a three-helix bundle by the movement of L1 and its interaction with H1 that lead to a new L1 structure with different regional electrostatic and hydrophobic surfaces, in comparison to PRL, that may contain the bioactive domain. Recombinant technology verified the proposed location of the bioactive domain by showing that a small protein comprising the H1 and L1 regions of human PRL is antiangiogenic.

## Results

### Tertiary structure of the vasoinhibin

The four-helix bundle conformation of PRL is maintained by non-covalent intermolecular interactions and by the central disulphide bond (C58-174) that covalently links L1 with H4 (Fig. 1a,b). Cathepsin D cleaves L3 connecting H3 with H4, which, after the reduction of the C58-174 disulphide bond, yields a 150 residue vasoinhibin^15^ (Fig. 1c). Cleavage and reduction removes H4 (Fig. 1d) and exposes the hydrophobic core of PRL (Fig. 1e), thereby suggesting an unstable, partially unfolded, pre-vasoinhibin conformation.

**Figure 2:**
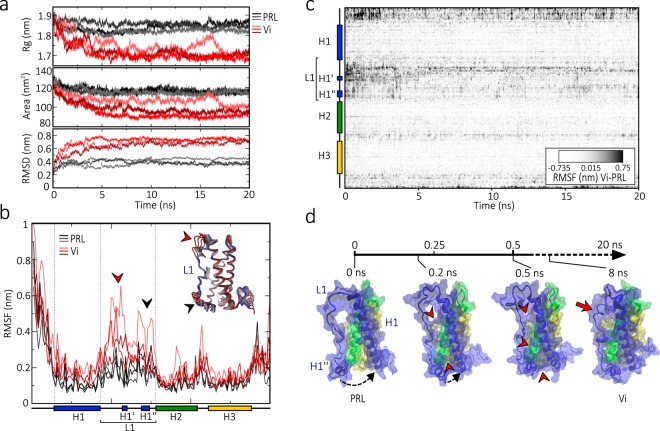
Tertiary structure of a 150 residue vasoinhibin (Vi). (**a**) Trajectory analysis of PRL and a Vi of 150 amino acids during 20 ns of molecular dynamic simulation (MD). Radius of gyration (Rg), surface area, and root mean square deviation (RMSD) of three MD simulations of PRL (dark lines) and Vi (red lines). (**b**) Total root mean square fluctuation (RMSF) per residue of Vi relative to PRL. The two major RMSF discrepancies, in first half (red arrowhead) and in the last half (black arrowhead) of the L1 are also illustrated in a sequentially superimposed tube model of the Vi trajectory indicating the regions of major movement in Vi compared to PRL. Red and blue colours represent changes from minimum (0 ns) and maximum (20 ns) MD, respectively. (**c**) Average RMSF per residue per time from triplicates obtained throughout the 20 ns MD of Vi relative to the respective average values of PRL. (**d**) Snapshots of the PRL to Vi transition through the MD simulation. The H1” movement towards H1 during first 0.2 ns (black arrow) and the interaction between residues in L1 and H1 starting at 0.2 ns (red arrowhead) are indicated. A helix-type structure in L1 is observed at ~8 ns and maintained until the end of the simulation (20 ns) (red arrow).

To investigate the structural changes leading to the stable vasoinhibin conformation, we performed a 20 ns MD simulation of a vasoinhibin comprising the first 150 residues of human PRL under physiological conditions of salt, temperature, and pH. Three different simulation runs were performed for vasoinhibin and for the full-length PRL under the same conditions. The trajectory analysis showed that removal of H4 causes the molecule to compress into a more compact structure than that of intact PRL (Fig. 2a). The radius of gyration (Rg) decreased in vasoinhibin and reached its minimal value at around 5 ns but was minimally altered in PRL. At 5 ns, the surface area of vasoinhibin was reduced relative to that of PRL and remained stable thereafter. The root-mean square deviation (RMSD) analysis showed that, while PRL was basically stable throughout the whole simulation, the RMSD of vasoinhibin increased dramatically during the first 5 ns and then remained without further change (Fig. 2a). The vasoinhibin instability essentially laid within the L1. The mobility of the protein residues analysed by the root-mean square fluctuation (RMSF) showed that the major discrepancies between vasoinhibin and PRL occurred precisely in the L1 region (Fig. 2b) and during the first 5 ns of the simulation, as indicated by the RMSF for each vasoinhibin residue per time normalized against the respective value in PRL (Fig. 2c). In addition, the N-terminal loop of vasoinhibin displayed strong normalized RMSF values in the first 2.5 ns of simulation (Fig. 2c).

Trajectory visualization showed that the compression of the molecule was driven by the immediate (<0.2 ns) movement of L1 towards H1, and that the contacts between both regions started from H1” up to the second half of L1 to eventually close the gap left by the removal of H4 (Fig. 2d). Hence, the rapid compression appears to be driven by hydrophobic forces from the exposed hydrophobic nuclei in vasoinhibin (Fig. 1e). Contacts with H1 were then extended to the first half of L1, which acquired new helix-like characteristics at ~8 ns (Fig. 2d). These conformational changes persisted at the end of the simulation, suggesting that vasoinhibin folds into a stable final structure at ~8 ns. This behaviour was observed at different force fields: CHARMM27 (Fig. 2), GROMOS96 54A7, and AMBER (Supplementary Fig. 1).

**Figure 3:**
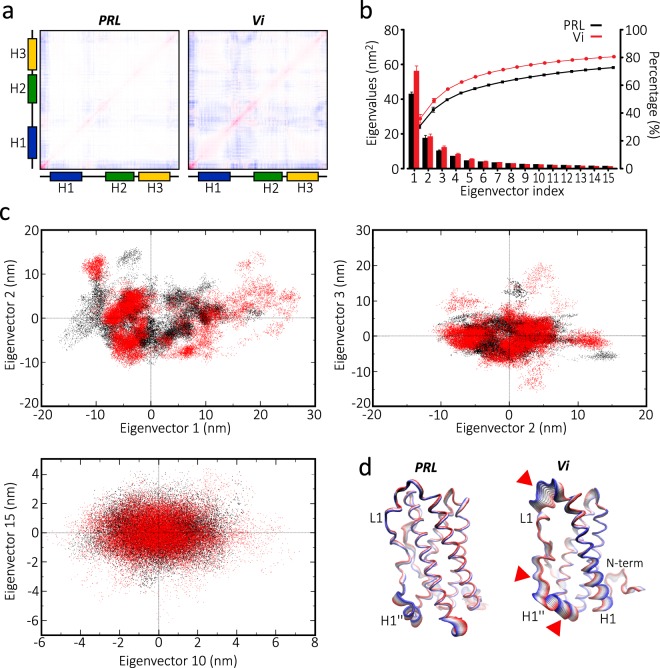
Principal component analysis (PCA) of trajectory of a 150 residue vasoinhibin (Vi). (**a**) Average backbone covariance matrix from PCA analysis of PRL and Vi. Highly correlated motions are in red and low correlation motions are in blue. Red intensity in the diagonal indicates the amplitude of fluctuations. (**b**) Bars show the first 15 eigenvectors of the covariance matrix of PRL and Vi (bars), whereas lines indicate the cumulative sum of the contribution to the total fluctuation percentage of the respective eigenvectors to each molecule. (**c**) The 2D eigenvector projections of the trajectory of PRL (black) and Vi (red). (**d**) Principal motion projected along the eigenvector 1 of PRL and Vi superimposed sequentially in 10 frames. Red and blue colours represent changes from minimum (0 ns) to maximum (20 ns) MD, respectively.

Principal component analysis (PCA) was then applied to the MD simulation trajectories in order to evaluate the global motions governing vasoinhibin conformational transition. PCA analysis confirmed that L1 is a highly fluctuating region and a major contributor to the collective motions of vasoinhibin. In contrast to PRL, the average backbone covariance matrix showed major correlated motions within vasoinhibin L1 and a low correlation with the rest of the molecule (Fig. 3a) implying the highly fluctuating independent movement of L1. Analysis of the first 15 eigenvectors with cumulative indexes indicated that the first eigenvector is responsible for most of the distinct molecular motion of vasoinhibin vs. PRL (Fig. 3b). The 2D projection of eigenvectors 1 and 2, 2 and 3, and 10 and 15 from each MD trajectory of vasoinhibin and PRL allowed visualization onto the essential space (Fig. 3c). As expected, global motions depicted by the two first components were greater and exhibited larger subspace dimensions in vasoinhibin compared to PRL. Finally, the subdomain motions within vasoinhibin and PRL were analysed by the corresponding motion mode of only the first eigenvector (Fig. 3d). The superimposed snapshots of the structures through 20 ns of simulation illustrate the dominant motions taking place along L1 of vasoinhibin relative to PRL (arrows).

### Secondary structure of the vasoinhibin

**Figure 4:**
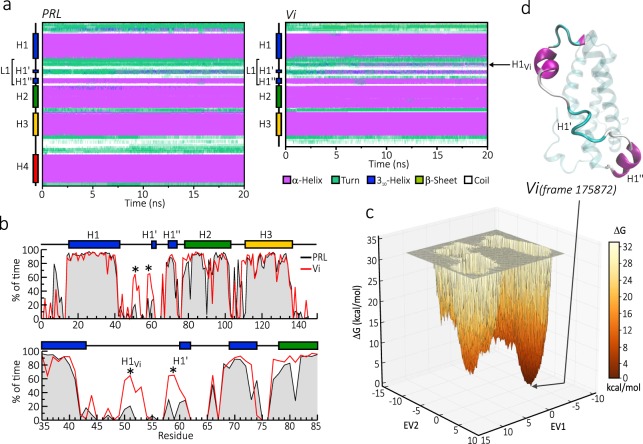
Dynamic changes of the secondary structure of a 150 residue vasoinhibin (Vi). (**a**) Changes of the secondary structure along the residue sequence of PRL (left) and the Vi (right) during 20 ns of molecular dynamic simulation (MD). The colours represent different secondary structures averaged from 3 independent MD. (**b**) Time percentage in helix conformation (helicity) per residue of PRL (black line) and Vi (red line) during a 200 ns MD simulation. Major discrepancies between PRL and Vi are indicated (*). (**c**) Free energy (DG) landscape (FEL) analysis of the Vi sampled throughout 200 ns of MD simulation. (**d**) Lowest energy structure obtained from FEL analysis.

Secondary structure analysis indicated that the α-helices of both PRL and vasoinhibin, maintained their stability throughout the 20 ns of MD simulation (Fig. 4a). However, early (~5 ns) in the simulation, the first region of the L1 in vasoinhibin organized into a new helix structure that fluctuated between a 3_10_-helix and α-helix (Fig. 4a). This helix was generated from a turn region and was defined as H1_Vi_ due to its exclusive presence in vasoinhibin and not in PRL (Fig. 4a). In addition, the H1’ in vasoinhibin, not in PRL, alternated with an α-helix conformation, which predominated at the end of the simulation. To better understand helical propensity, we compared the percentage of time in secondary structure for each amino acid between vasoinhibin and PRL in a simulation extended to 200 ns (Fig. 4b). As expected, the main differences occurred in the L1 residues 50 to 53 and 58 to 61 comprising H1_Vi_ and H1’, respectively. These helices were only present or better preserved in vasoinhibin relative to PRL.

Finally, we used free energy landscape analysis to identify the natively folded vasoinhibin structure, which is defined as the conformation requiring minimal energy (Fig. 4c,d). The free energy landscape representation was achieved by projecting the trajectories of the first two principal components of motion, eigenvectors 1 and 2 (EV1 and EV2) (Fig. 3), as reaction-coordinates (Fig. 4c). From this analysis, the structure in frame 175872 corresponded to the overall minimal-energy vasoinhibin conformation in the whole 200 ns simulation (Fig. 4d). It contained a well-formed H1_Vi_ but an unfolded H1’.

Both PRL and vasoinhibin show a kink in H2 due to a proline in position 94 (P94) which disrupts and bends H2 between a serine at position 90 (S90) and P94 (Supplementary Fig. 2). The kink in PRL (pdb 1RW5) is less evident, it has an angle of ~14° with a maximal bending point at S90; while in vasoinhibin the angle is ~33 °C with maximal bending point at P94 (Supplementary Fig. 2a,b). The larger angle in vasoinhibin may result from the reduced constraint imposed by the absence of H4. Despite a larger H2 kink, the H2 intercrossing angle with H1 does not change in vasoinhibin (40.2°) relative to PRL (37.3°) (Supplementary Fig. 2c), but the intercrossing angle between H2 and H3 does increase in vasoinhibin (35.5° and 20.6° for vasoinhibin and PRL, respectively) (Supplementary Fig. 2d).

### Solvent accessible surface area of the vasoinhibin

**Figure 5:**
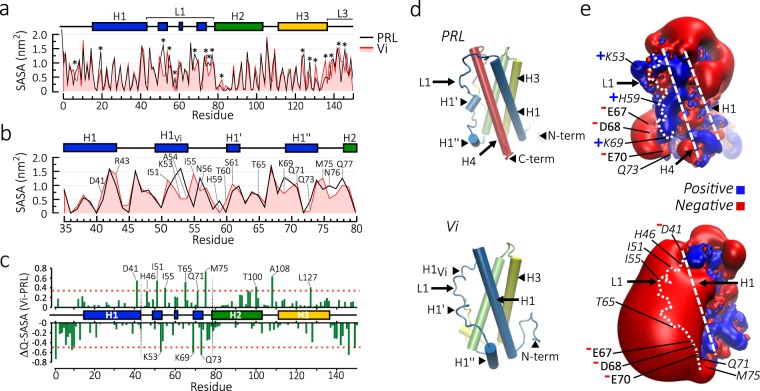
Solvent accessible surface area (SASA) and electrostatic surface potential of a 150 residue vasoinhibin (Vi). (**a**) Average SASA (nm^2^) per residue of PRL and Vi in a 200 ns molecular dynamic simulation (MD). SASA discrepancies between PRL and Vi are indicated (*). (**b**) Average SASA values in the L1 region. Residues with major SASA differences are indicated. (**c**) Differential SASA coefficient (ΔQ-SASA) of the minimal-energy Vi vs. PRL. Positive and negative values indicate residues that are more or less exposed, respectively, in Vi compared to PRL. Residues above or below the 90^*th*^ percentile of ΔQ-SASA (pointed red line) are considered significantly more exposed or buried, respectively. (**d**) Representation of the lowest energy structure of PRL and Vi. (**e**) Positive and negative electrostatic potential isosurface (contour level of ±2 KT e^−1^) depicted in blue and red colours, respectively, of the PRL and Vi shown in (**d**). Position of H1, L1 and H4 are indicated by white dashed lines. Residues with major SASA discrepancies and their positive (+) and negative (−) charges are indicated.

Solvent exposure of active residues is important for peptide activity^18^ and regional differences in solvent exposure between vasoinhibin and PRL may help localize the active domain of vasoinhibin. Solvent accessible surface area (SASA) analysis of each protein residue throughout a 200 ns simulation showed major discrepancies between vasoinhibin and PRL in the L1, H3, and L3 regions (Fig. 5a). Because the differing residues in H3 and L3 are absent in a bioactive vasoinhibin comprising the first 123 amino acids of human PRL, we focused on SASA differences located in the L1, a major determinant of the stable conformation of vasoinhibin (Fig. 5b). Residues I51, I55, T60, S61, T65, Q71, and M75 were more exposed to solvent, whereas residues K53, N56, K69, Q73, N76, and Q77 were less exposed throughout the whole simulation of vasoinhibin relative to PRL (Fig. 5b). The differential SASA coefficient (ΔQ-SASA) obtained by subtracting the SASA values of PRL (PDB 1RW5) from those of the minimal energy conformation of vasoinhibin (frame 175872, Fig. 5d) indicated that in the H1 and L1 regions, the amino acids D41, H46, I51, I55, T65, Q71, and M75 were more solvent exposed, whereas the amino acids K53, K69 and Q73 were less solvent exposed in vasoinhibin than in PRL (Fig. 5c).

Some residues in the N-terminal loop of vasoinhibin were less exposed to solvent than in PRL (Fig. 5c), suggesting a more stable region. This is consistent with the strong normalized RMSF values observed in the N-terminal loop of vasoinhibin during the first 2.5 ns of simulation, which was followed by a more stable RMSF (Fig. 2c). Also, residues in the second half of H2 progressively increased their relative SASA values (Fig. 5c), indicating that removal of H4 in vasoinhibin increases their solvent exposure.

### Electrostatic surface potential of the vasoinhibin

The differentially exposed residues of vasoinhibin modified the electrostatic properties of its surface relative to PRL. Figure 5e shows the color-coded computer graphic representation of the electrostatic potential throughout the surface of both proteins in their native conformation. Loss of H4 led to a dominant negative potential at the L1 and H1 regions of vasoinhibin. The stronger negative surface covered almost half of vasoinhibin and had its centre located at the L1 region, whereas the rest of the molecule showed little change (Fig. 5e). This highly negative surface potential region appears to be largely influenced by the burial of the basic residues (K53 and K69), since the acidic residues within the L1 (D41, E67, D68, and E70) remained solvent exposed. Because charged residues buried within the protein must make specific ‘salt-bridge’ charge pairs, we performed a computational analysis of salt-bridges in PRL and vasoinhibin using the ESBRI web server^19^ (Supplementary Fig. 3). PRL contained intramolecular salt-bridges most of which were located within lateral structures (loops and α-helices), except for R164-E101 found in the core of the molecule. Residues K53 and K69, buried in vasoinhibin, were located in L1 and formed salt-bridges with E93 in H2 and D20 in H1, respectively (Supplementary Fig. 3a). Analysis of the minimal distance between paired residues showed that K53-E93 is less stable than K69-D20 (Supplementary Fig. 3b).

### Molecular hydrophobic potential of the vasoinhibin surface

**Figure 6:**
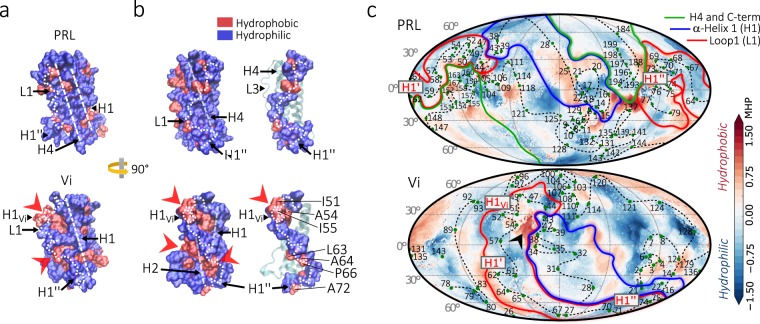
Hydrophobic surface potential of a 150 residue vasoinhibin (Vi). (**a**) Hydrophilic (blue) and hydrophobic (red) residues of PRL and Vi shown in a surface representation model. Changes in the exposure of hydrophobic residues in Vi relative to PRL are indicated (red arrowheads). The localization of secondary structures (H1, L1, and H4) is indicated with white dashed lines. (**b**) Lateral view of the whole molecule (left) or the L1 region (right) using the surface. (**c**) Protein surface topography representation of molecular hydrophobic potential of PRL and Vi. Some exposed amino acids are indicated (green dots) and connected sequentially (black dotted line). Colour surface patches represent secondary-structure regions of PRL and Vi. Secondary structures within L1 (H1_*Vi*_, H1’ and H1”) are indicated (boxes). A highly hydrophobic patch in Vi is indicated (black arrowhead).

Hydrophobicity is a relevant force influencing the propensity and strength of protein-protein interactions^18^. At the H1-L1 region, more hydrophobic residues were exposed on the surface of vasoinhibin than of PRL (Fig. 6a,b). Therefore, the H1-L1 region may be an active spot for the formation of vasoinhibin-protein complex driving its biological activity. Hydrophobic spots include H1_Vi_ and a region near H1” where hydrophobic residues I51, A54, and I55, and L63, A64, P66, and A72 are exposed, respectively (Fig. 6b).

For a more descriptive assessment of the hydrophobic properties of vasoinhibin relative to PRL, we calculated the molecular hydrophobic potential^20^ of both proteins and projected them into a 2-dimension spherical map using the protein surface topography method^21^ (Fig. 6c). In PRL, the surface of H4 shares borders with H1 and L1, but H1 and L1 do not contact each other, except at the level of the H1 transition into L1. In contrast, in vasoinhibin, the H1 and L1 regions have a long region of contact throughout their surfaces. This long border is the major distinct structural feature between vasoinhibin and PRL and may thereby contribute to the putative bioactive domain of vasoinhibin. Consistent with this possibility, a highly hydrophobic spot was observed precisely in the border between H1 and L1 at the level of H1_Vi_ (Fig. 6c).

Altogether, MD simulation identified the L1 region as the distinctive structural feature in vasoinhibin, which may correspond to the bioactive domain. To help validate this possibility, we investigated the antiangiogenic properties of a recombinant protein containing the first 79 amino acids of human PRL comprising only the H1 and L1 regions.

### A protein comprising residues 1 to 79 of human PRL is antiangiogenic

**Figure 7:**
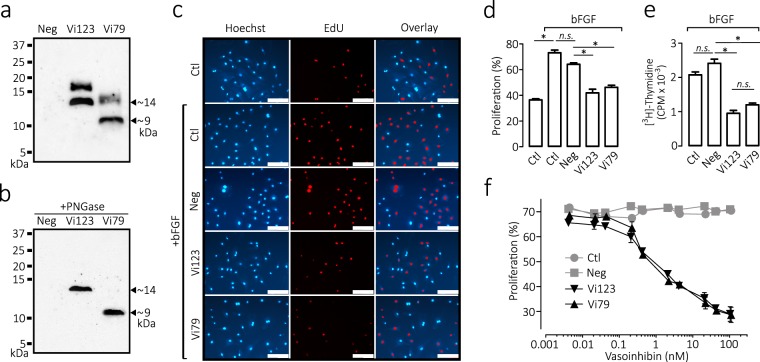
A recombinant protein comprising amino acids 1 to 79 of human PRL inhibits the proliferation of endothelial cells. (**a**) Western blot analysis of the conditioned media (CM) obtained from HEK293T/17 cells stably transduced with lentiviral vectors encoding the first 79 residues (~9 kDa) or the first 123 residues (~14 kDa, a control vasoinhibin) of human PRL. The conditioned media of HEK293T/17 cells transduced with the empty lentiviral vector was the negative control (Neg). (**b**) Western blot of the same conditioned media shown in (**a**) after treatment with Peptide N-glycosidase F (PNGase). Numbers on left indicate the molecular weight of marker proteins. (**c**) Representative fields of bovine pulmonary artery endothelial cells (CPAE) stained for DNA (Hoechst), DNA synthesis (EdU), and the overlay of both reactions. Cells were incubated for 24 h with or without basic fibroblast growth factor (bFGF) in combination or not (Ctl) with conditioned media without (Neg) or with the 79- (Vi79) or the 123- (Vi123) residue vasoinhibins. Scale bar, 100 *μ*m. (**d**) Cell proliferation was quantified by the Edu-click reaction and expressed relative to the total number of cells in the field. Values are means ± SEM of 3 independent experiments (*p < 0.001). (**e**) Cell proliferation was evaluated by the [^3^H]-thymidine incorporation to DNA in CPAE incubated for 24 h with bFGF together or not (Ctl) with conditioned media lacking (Neg) or containing with the Vi79 or the Vi123 residue vasoinhibins. Values are means ± SEM of triplicate determinations (*p < 0.001). (**f**) Dose-dependent inhibition of bFGF-induced proliferation of bovine umbilical vein endothelial cells (BUVEC) incubated for 24 h with bFGF in combination or not (Ctl) with conditioned media without (Neg) or with the Vi79 or the Vi123. Cell proliferation was quantified by the Edu-click reaction and expressed relative to the total number of cells in the field. Values are means ± SEM of triplicate determinations.

HEK 293 T/17 cells were stably transfected with one of three lentiviral vectors: an empty vector (negative control), a vector encoding the first 123 residues of human PRL (control vasoinhibin), or a vector encoding the first 79 residues of PRL. The conditioned media of the transfected cells showed immunoreactive bands with the expected molecular weights of 9 and 14 kDa for the 79- and 123-amino acid proteins, respectively (Fig. 7a). In addition, there were higher molecular weight immunoreactive PRL-like proteins of 14 and 18 kDa in the conditioned media containing the 79- and the 123-residue proteins, respectively, which correspond to glycosylated isoforms of the recombinant proteins. PRL has a N-glycosylation site at asparagine 31 (N31) and only the expected 9 and 14 kDa proteins remained upon digestion of the respective conditioned medium with peptidyl N-glycosidase F (PNGase F), which cleaves N-linked oligosaccharides (Fig. 7b). No immunoreactive proteins were detected in the conditioned medium of cells transfected with the empty lentiviral vector (Fig. 7a,b). Also, there was no PRL-like immunoreactive material in the upper section of the Western blots that could reflect the presence of PRL fragment aggregates (Supplementary Fig. 4). However, non-reducing Western blots showed that the 79- and 123-PRL fragments in the conditioned media formed disulfide bond dimers of ~18 kDa and ~28 kDa of the 79- and 123-residue PRLs, respectively (Supplementary Fig. 6). The 79-amino acid fragment was only detected as a dimer, whereas equivalent amounts of dimer and monomer occurred for the 123-residue PRL (Supplementary Fig. 6b). Disulfide bond aggregates were expected due to the presence of a free SH group (from C58) created by the cleavage of PRL.

Vasoinhibin, including the one comprising the first 123 residues of human PRL, inhibits the proliferation^22^ and migration^23^ of endothelial cells. To determine whether the 79-amino acid PRL is antiangiogenic, the conditioned media containing equivalent amounts of the 79- or the 123-amino acid proteins were added to bovine pulmonary artery endothelial cells (CPAE) in culture and their effect on DNA synthesis was measured by the incorporation of 5-ethynyl-2′-deoxyuridine (EdU) and the click reaction (Fig. 7c). The conditioned media containing the 79- or the 123-residue proteins inhibited basic fibroblast growth factor-induced CPAE proliferation compared to the conditioned medium from the negative group or to cells not treated with conditioned medium (control) (Fig. 7d). In support of these findings, the negative control using the conditioned medium of HEK293T/17 stably transduced with lentiviral vectors encoding full-length PRL was inactive, in spite of containing over 20-times more PRL protein than the amount of PRL fragments produced from 79- and 123-residue encoding lentiviral vectors (Supplementary Fig. 7a,b). Also, the total protein level and pattern revealed by Coomassie blue- and Silver-stained gels were similar among the different conditioned media (Supplementary Fig. 7c,d), indicating that an excess of a non-antiangiogenic protein (PRL) and total protein differences did not influence the results. Similar effects on CPAE proliferation were also observed by measuring ^3^H-thymidine incorporation (Fig. 7e). Likewise, the 79- and the 123-residue proteins inhibited the proliferation of bovine umbilical vein endothelial cells (BUVEC), assayed by the click reaction, in a concentration-dependent manner and with similar potency (Fig. 7f).

**Figure 8:**
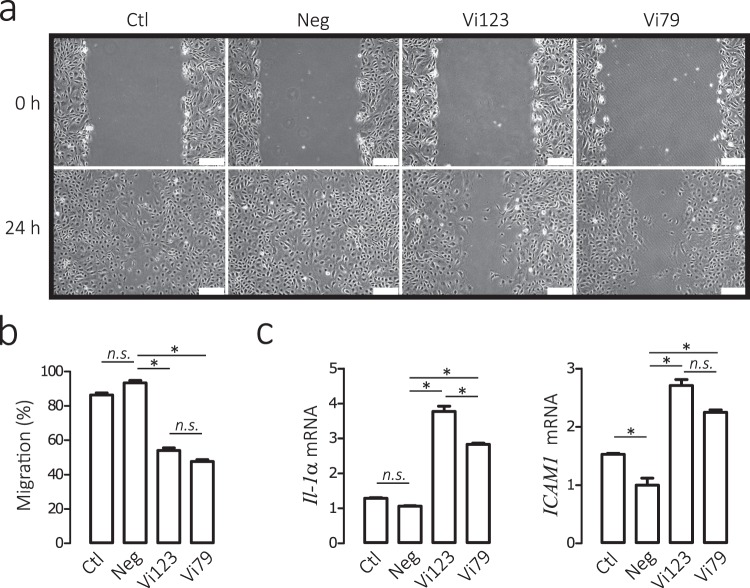
A recombinant protein comprising amino acids 1 to 79 of human PRL inhibits the migration of endothelial cells and the endothelial cell expression of vasoinhibin target genes. (**a**) Bovine pulmonary artery endothelial cell (CPAE) monolayers were scratched and treated for 24 h with medium alone (Ctl) or containing transduced HEK293T/17 conditioned media without (Neg) or with the Vi79 or the Vi123 residue vasoinhibins. Representative images of wound sealing at 0 and 24 h. Scale bar, 100 *μ*m. (**b**) The level of migration into the wound scratch was quantified as the percentage of wound sealing at 24 h relative to respective group value at onset (0 h). Values are means ± SEM of 3 independent experiments (*p < 0.001). (**c**) Expression of vasoinhibin target genes interleukin-1α (*IL1A*) and intercellular adhesion molecule 1 (*ICAM1*) measured by quantitative real-time PCR (qRT-PCR) normalized against cyclophilin A (PPIA). CPAE were treated for 4 h with medium alone (Ctl) or containing conditioned media without (Neg) or with Vi79 or Vi123.Values are means ± SEM of 3 independent experiments (*p < 0.001).

To evaluate actions on endothelial cell migration, we employed the well-established wound-healing assay^24^, in which a wound scratch is introduced in a confluent CPAE monolayer to stimulate migration. The CPAE in the negative and control groups sealed ≈80% of the wound 24 h after wound scratch, whereas the cells treated with the 79- and 123-residue proteins only showed ≈50% sealing at this time (Fig. 8a,b). Finally, IL1A and ICAM1 are target genes of vasoinhibin in endothelial cells^25^, and both genes were upregulated in CPAE treated with the conditioned media containing the recombinant proteins, but not in the cells under the negative and control conditions (Fig. 8c).

**Figure 9:**
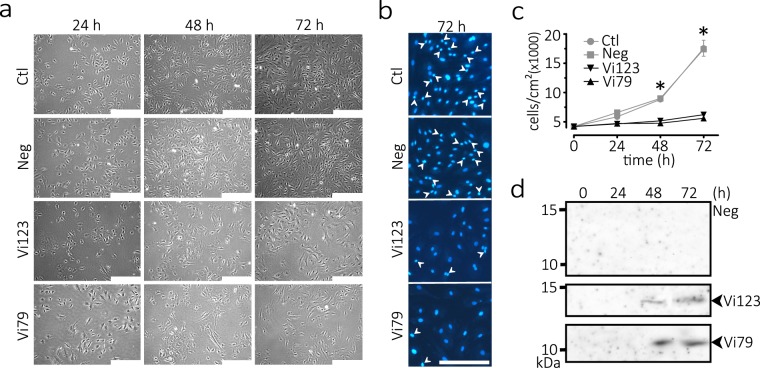
Proliferation of endothelial cells producing a 79 amino acid vasoinhibin. (**a**) Representative fields of bovine umbilical vein endothelial cells (BUVEC) transduced or not (Ctl) for 24, 48, or 72 h with lentiviral vectors coding for the 79- and 123-residue vasoinhibins (Vi) or with the empty vector (Neg). Scale bar, 500 *μ*m. (**b**) Proliferation is further illustrated by the number of small and brilliant Hoechst-stained nuclei (arrowheads) characteristic of dividing cells at 72 h post-lentiviral transduction. Scale bar, 250 *μ*m. (**c**) BUVEC density following 24, 48 and 72 h of lentiviral transduction. Values are means ± SEM of 3 independent experiments (*p < 0.001 vs. Ctl and Neg). (**d**) Western blot analysis of BUVEC conditioned media at 0, 24, 48, and 72 h after transduction with lentiviral vectors coding for Vi79 (~9 kDa) and Vi123 (~14 kDa) or the empty vector (Neg).

To further document the antiangiogenic effect of the 79 residue vasoinhibin, we compared the proliferation of non-transduced BUVEC with that of BUVEC transduced with the lentiviral vectors coding for the 79- and 123-residue vasoinhibins or with the empty vector (Fig. 9). BUVEC transduced with the 79- and 123-residue vasoinhibin vectors showed a significant reduction in their proliferation rate at 48 and 72 h after transduction (Fig. 9a–c), which correlates with the accumulation of the respective vasoinhibin isoform in the BUVEC conditioned medium (Fig. 9d, Supplementary Fig. 5). No vasoinhibin-like immunoreactivity was detected in the negative control transduced with the empty vector (Fig. 9d, Supplementary Fig. 5). Moreover, increased proliferation, indicated by the higher number of small and brilliant Hoechst-stained nuclei was evident in the control groups compared with BUVEC expressing the 79- and 123-residue vasoinhibins (Fig. 9b).

### MD simulation of vasoinhibins of 79 and 123 residues

**Figure 10:**
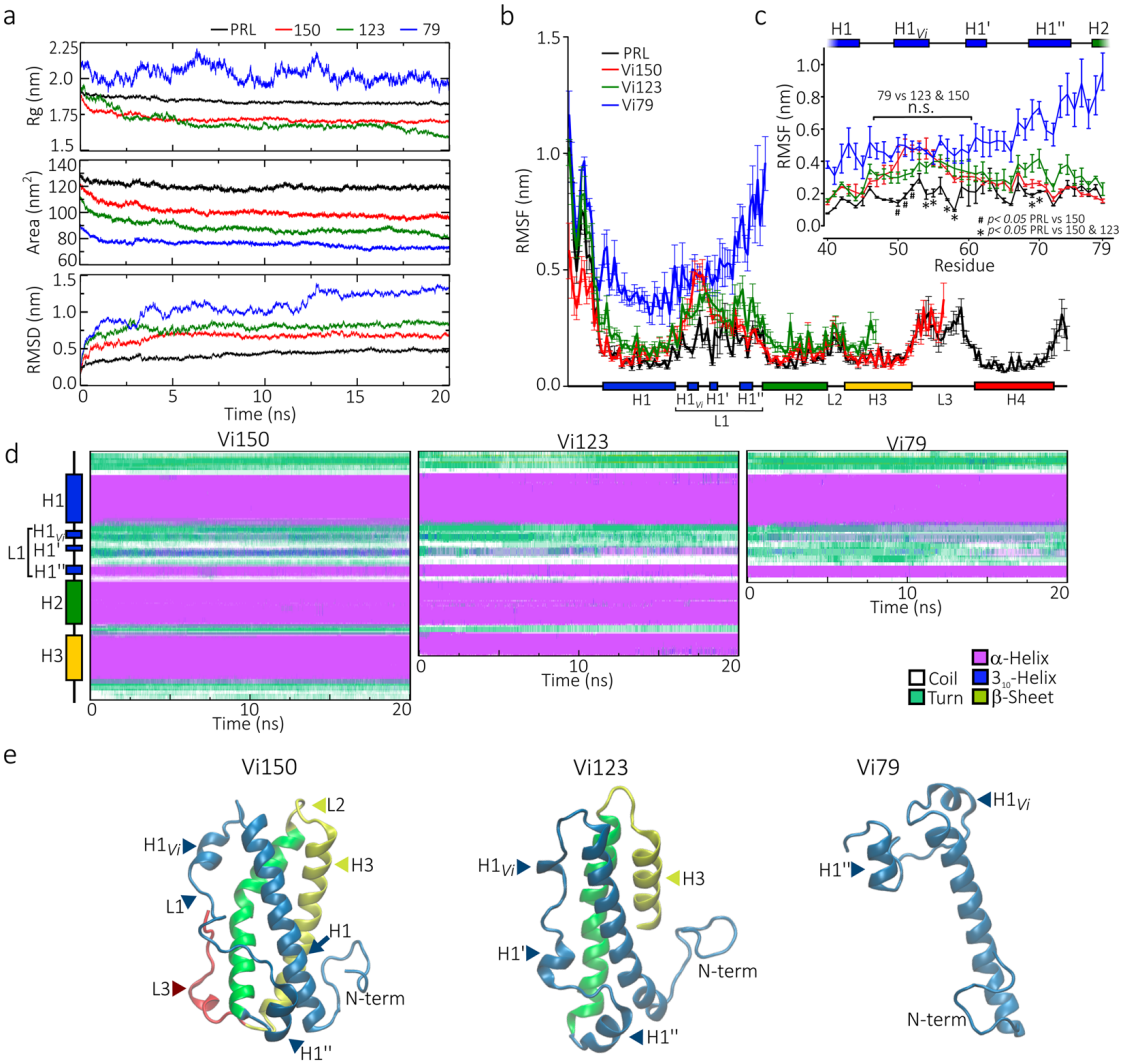
Comparison of the molecular dynamic simulation (MD) of the 150, 123, and 79 amino acid vasoinhibins. (**a**) Trajectory analysis of PRL and vasoinhibins of 150, 123 and 79 amino acids (Vi150, Vi123, and Vi79, respectively) during 20 ns of MD simulation. Average radius of gyration (Rg), surface area, and root mean square deviation (RMSD) of three MD simulations per molecule. (**b**) Mean ± SEM root mean square fluctuation (RMSF) per residue of three independent MD simulations. (**c**) RMSF values in the L1 region. Values of each PRL residue are statistically different (p < 0.05) from both Vi150 and Vi123 (*), or only from Vi150 (^#^) as indicated. The horizontal bracket indicates the region of L1 in which all three vasoinhibins display similar RMSF values (residues 47 to 60). (**d**) Dynamic changes of the secondary structure profile per residue of Vi150, Vi123, and Vi79 throughout 20 ns of MD simulation. Average composition of at least 3 independent MD simulations. (**e**) Representative structures of Vi150, Vi123, and Vi79 at the end of the 20 ns MD simulation. Structural motifs are indicated.

In search of the functional structural denominator, we performed MD simulation of the vasoinhibins comprising amino acids 1 to 79 and 1 to 123 of PRL and compared them to that of the 150 residues vasoinhibin. MD results were very similar between the vasoinhibins of 123 and 150 residues. Both vasoinhibins showed an immediate (5 ns) molecular compression, surface area reduction, and enhanced RMSD values, which stabilized thereafter (Fig. 10a, Supplementary Fig. 8). Also, similar to the 150 amino acid vasoinhibin, the 123 residues vasoinhibin showed major discrepancies in mobility of protein residues (RMSF) relative to PRL in the first part of L1 (Fig. 10b); the three α-helices maintained their stability throughout the simulation, and L1 exhibited increased helix propensity (with H1_Vi_ being smaller and less stable and H1’ more constant) (Fig. 10d,e, Supplementary Fig. 8). In contrast, the structure of the 79 amino acid vasoinhibin was less compacted and highly mobile. Its Rg, RMSD, and RMSF values were higher and had larger fluctuations, reached a more compact state after the first 20 ns simulation, and then a less compressed, unstable structure at ~150 ns (Fig. 10a, Supplementary Fig. 8). Nevertheless, in the first part of L1 (residues 47 to 60) the RMSF values of all three vasoinhibins (79, 123, and 150 residues) were similar to each other and different from PRL (Fig. 10b,c). Also, in spite of its high mobility and disorganized state, L1 maintained helix propensity and a stable H1_Vi_ overtime (Fig. 10d,e; Supplementary Fig. 8).

## Discussion

The PRL/vasoinhibin axis is a defined endocrine axis in which the generation of vasoinhibin is regulated by the hypothalamo, the pituitary, and the target tissue levels^7^. This axis regulates the angiogenesis required for the growth and function of reproductive and non-reproductive organs and contributes to the pathogenesis of angiogenesis-related retinal and cardiac diseases and diseases occurring during and after pregnancy^10,26,27^. Moreover, the conversion of PRL to vasoinhibin is the basis of two clinical trials in which vasoinhibin is the target of therapeutic interventions^28^. However, the molecular transition of PRL to vasoinhibin is poorly understood and there is no information regarding the solution structure of vasoinhibin. Improving this understanding could lead to quantitative assays specific for vasoinhibin and to the generation of agonists and antagonists for the treatment of disease. Here, we have modeled the structure of vasoinhibin by MD simulation and show that it can acquire a stable three-helix bundle conformation due to the movement of L1 and its interaction with H1. The newly created H1 and L1 molecular surfaces display solvent accessible, electrostatic, and hydrophobic properties that are distinct from uncleaved PRL and may correspond to the vasoinhibin antiangiogenic domain. Consistent with this hypothesis, we show that a recombinant protein comprising only the H1 and L1 regions of PRL has antiangiogenic properties.

A three-helix bundle conformation occurrs ubiquitously in proteins with high-affinity interactions such as enzymes, enzyme inhibitors, and transcription factors^29^. The three-helix bundle conformation in a 150 residue vasoinhibin is led by an immediate molecular compression observed at all MD simulations and under different force fields (GROMOS96 54A7, AMBER and CHARMM27). This suggests that a robust physical property, such as hydrophobicity^30^, influences the transition and stabilization of vasoinhibin. Indeed, removal of H4 exposes the hydrophobic nuclei of PRL, which is immediately buried again by the movement of L1 and the contacts of L1 with H1. In addition, L1 displays a higher α-helix propensity and generates a new α-helix (H1_Vi_). These structural changes govern the vasoinhibin conformational transition and persist in the minimal energy native vasoinhibin.

Residues within the H1 and L1 regions of vasoinhibin show major discrepancies in their solvent accessible surface relative to PRL. Also, a dominant negative surface area prevails at the L1 and H1 regions of vasoinhibin that is largely influenced by the burial of basic residues not occurring in PRL. Finally, more hydrophobic residues are exposed in vasoinhibin and create hydrophobic spots in the border between H1 and L1 and at the level of H1_Vi_. Because solvent exposure of active residues, polar interactions, and hydrophobicity influence protein-protein interactions required for bioactivity^31^, we reasoned that the altered H1 and L1 regions generated upon PRL cleavage corresponds to the vasoinhibin antiangiogenic domain. Supporting this possibility, the first 79 residues comprising the H1 and L1 regions of human PRL are sufficient to excert antiangiogenic effects.

Antiangiogenesis is demonstrated by the inhibition of the proliferation and migration of endothelial cells in culture. The antiangiogenic activity of the 79 residue recombinant protein is of similar potency (EC_50_ = 1 nM) to that of a conventional 123 residue vasoinhibin encompassing the H1, L1, H2, L2, and part of H3 regions of human PRL and consistent with the vasoinhibin potency reported previously^22^. Glycosylation and disulfide bond dimerization of the PRL fragments may not influence their bioactivity. The antiangiogenic activity of the 79- and 123-residue vasoinhibins, containing the glycosylated isoforms and the disulfide dimers, is of similar potency (EC_50_ = 1 nM) to that of a non-glycosylated 123-residue vasoinhibin produced in E.coli where C58 was mutated to serine to avoid incorrect intra-molecular and inter-molecular disulfide bond formation leading to aggregation^22^.

The antiangiogenic action of the 79-residue protein verifies that the vasoinhibin active domain lies within the L1 and H1 regions of PRL. The fact that the 79-amino acid vasoinhibin is in a dimeric form implies that the active domains continue to be functional in spite of dimerization. The structural basis of vasoinhibin dimer formation is unclear. While the intermolecular disulfide bond linking the two cysteines at position 58 is a key component, other interactions are likely involved. Aggrescan3D^32^ and Tango^33^ software predict a “hot spot” for aggregation within residues 18 to 29 of H1 (Supplementary Fig. 6c) that may enforce the hydrophobic forces pairing two H1. Also, in PRL, histidine 27 forms a salt-bridge with asparagine 183 in H4 (Supplementary Fig. 3a) and, in the absence of H4, the unpaired histidine 27 may form salt-bridges across the vasoinhibin dimer. Moreover, metal ion-dependent motifs involving histidine 127 create a metal binding site in PRL that is responsible for Zn-dependent PRL aggregation^34,35^. We propose a model of dimer assembly in which two H1 are paired in a parallel manner and both L1 are linked by the cysteine 58 intermolecular disulfide bond (Supplementary Fig. 6d). This model is consistent with the interactions proposed above and with the exposure and functionality of the two bioactive domains within the vasoinhibin dimer, but should be tested experimentally.

 Our findings are consistent with previous observations showing that all PRL fragments with vasoinhibin properties share the N-terminal end of PRL, whereas C-terminal PRL fragments are devoid of antiangiogenic effects^7,36^. However, the H1 and L1 primary structure by itself is not responsible for vasoinhibin activity, since this sequence is present in uncleaved PRL lacking antiangiogenic action. Rather, a structural conformation within the H1 and L1 regions emerges upon proteolytic cleavage and accounts for antiangiogenesis.

To gain insights into the vasoinhibin bioactive structural domain, we performed MD simulation in the 123- and 79-residue vasoinhibins. A comparative analysis revealed structural similarities between the vasoinhibins of 123 and 150 amino acids. Both compress into a three-helix bundle by the movement of L1 and its interaction with H1 and share a similar secondary structure. L1 helical propensity relative to PRL is also higher in the 123-residue vasoinhibin, which displays a smaller and unstable H1_Vi_ and a more constant H1’ than the 150 amino acid vasoinhibin. In contrast, and as expected, the molecular conformation of the vasoinhibin of 79 residues is different. It has a less compacted unfolded structure and an elevated global motion largely due to the high mobility and disorganized state of L1. Nevertheless, the mobility of the protein residues in the first part of L1 (residues 47 to 60) is similar among the three vasoinhibin isoforms with 79, 123 and 150 residues and distinct from PRL. Also, L1 shows increased helical propensity and forms a new H1_Vi_ helix in the three vasoinhibins. In PRL, the C58-174 disulfide bond between L1 and H4 restricts the mobility of L1 holding it against the four α-helix bundle and conferring a more compact and stable configuration^14^. Cleavage of PRL without removal of the C58-174 bond does not result in the acquisition of vasoinhibin properties^37^. Loss of H4 in the vasoinhibin must occur for L1 to acquire the mobile configuration and high helical propensity. We conclude that these L1 conformational changes are major structural denominators of vasoinhibin determining its biological properties.

Consistent with this notion, a peptide sequence of 14 amino acids (residues 45 to 58) located in the early part of L1 of buffalo PRL exhibits antiangiogenic effects^38^. This sequence was unveiled due to its 35.7% homology with human somatostatin, a known antiangiogenic factor^39^. However, it has also been claimed that the vasoinhibin active domain is located at H2, specifically in a 14 amino acid sequence (residues 80 to 93) with “tilted peptide” characteristics^40^. Tilted peptides are short peptides with an asymmetric distribution of their hydrophobic residues when helical. When synthesized in fusion with maltose-binding protein to improve solubility, the tilted sequence of human PRL inhibits angiogenesis. However, its potency is low (4- and 32-fold less than that of a vasoinhibin) and a vasoinhibin mutated to abolish the structural characteristics of the tilted sequence only shows a partial reduction of bioactivity^40^. In PRL, the tilted peptide in H2 is buried by the three other α-helices and the third loop and it is unclear whether proteolytic cleavage uncovers such sequence. In this regard, MD simulation of the 150 residue vasoinhibin revealed that only the residues in the second half of H2, which do not contain the tilted sequence, are more solvent exposed than in PRL. While it is possible that the antiangiogenic action of vasoinhibin involves several functional epitopes, the fact that the 79-residue protein has effects similar to those of larger vasoinhibins indicates that the main vasoactive domain lies within H1 and L1 and that domains beyond these regions are not essential for antiangiogenesis.

Besides angiogenesis, vasoinhibin acts on endothelial cells to regulate other important aspects of vascular physiology. It promotes the expression of various chemokines and endothelial cell ahesion molecules including ICAM1 and IL1A to increase leukocyte extravasation^25^. The fact that the 79 kDa vasoinhibin induces the expression of ICAM1 and IL1A in endothelial cells implies that the antiangiogenic domain may also be responsible for this action. Other vascular-related effects of vasoinhibin include the inhibition of the endothelial nitric oxide synthase (eNOS) activation to reduce vasodilation and vasopermeability^41,42^ and the binding to plasminogen activator inhibitor 1 to promote fibrinolysis^8^. Furthermore, vasoinhibin has non-vascular actions. It acts on retinal pigment epithelial cells to inhibit outer blood retinal barrier permeability^43^; binds to lung fibroblast to stimulate inflammatory reactions^44^; and affects the nervous system by triggering neuroendocrine responses^45^, promoting behaviors associated with anxiety and depression^46^, and acting directly on neurons to inhibit neurite outgrowth^47^. It remains to be determined whether all vascular and non-vascular effects involve the same antiangiogenic domain of the molecule. Unveiling the residues conforming the active site(s) of vasoinhibin should be useful to identify functional interactions with known and new binding partners and receptors, thereby assisting in understanding the molecular mechanisms mediating its biological actions.

The molecular heterogeneity of vasoinhibin depends on the various proteases that cleave the PRL molecule at different sites and at several cellular and tissue contexts. Cathepsin D, matrix metalloproteases (MMP), and bone morphogenetic protein 1 generate PRL fragments ranging from the first 80 to the first 159 residues^7^. Endogenous PRL fragments of similar sizes have been detected in several rodent and human tissues and body fluids^7^, but only the biological activity of PRL fragments containing the first 123 to 159 residues (14 to 18 kDa) have been investigated and identified as vasoinhibins^6,7^. Here, we extend the vasoinhibin properties to a 79 residue PRL having a 9 kDa molecular mass. A similar PRL fragment is generated by matrix metalloproteases^16^ and is present in the amniotic fluid of pre-eclamptic women^13^ implying that it corresponds to an active member of the vasoinhibin family.

In summary, our findings extend the vasoinhibin family to smaller proteins and provide important structural information for the development of more potent agonists and antagonists. The MD results locate a potential similar structure to that of the bioactive domain of vasoinhibin within the first half of L1. The structure and folding properties of vasoinhibin warrant further investigation using a variety of experimental techniques (circular dichroism, size-exclusion chromatography, nuclear magnetic resonance spectroscopy, and X-ray crystallography). Studies in progress are identifying key surface residues within the putative bioactive region that when mutated alter the formation of binding complexes and biological properties. This information will impact the generation of potent vasoinhibin agonists and antagonists and the development of specific immunoassays for quantifying the levels of the proteins in the clinic.

## Methods

### Molecular Dynamic (MD) simulation

The coordinates of soluble human PRL were downloaded from the Protein Data Bank (PDB entry 1RW5) were as reported^14^. The C-terminal fragment of PRL was removed in order to generate the pre-vasoinhibins comprising residues 1 to 150, 1 to 123, and 1 to 79. Classical MD simulation was performed for PRL and the vasoinhibins using the GROMACS 5.1 package^48,49^. The CHARMM27 all-atom force field was used to generate the topology^50^. Simulations were carried out at neutral pH, in which protonation states and tautomerization of histidines were determined by the H^++^ ^51^ (http://biophysics.cs.vt.edu/H++) and the PROTOSS^52^ (http://proteinsplus.zbh.uni-hamburg.de/) web servers, respectively. Proteins were solvated using the TIP3P water model^53,54^ in a cubic box under the periodic boundary conditions and a distance of 1.0 nm from the protein to the surface of the box. The system was neutralized by the addition of counter ions. A salt concentration of 120 mM was maintained by 50 Na^+^ and 50 Cl^*−*^ ions added into a 700.53 nm^3^ box. The energy of the system was minimized using the steep descent integrator with no constraints for 1000 steps and equilibrated for 100 ps at a temperature of 310 K generated by the canonical ensemble NVT. Pressure and density were maintained using the isobaric-isothermal ensemble NPT. Equilibrium steps were performed applying position restraints to the molecule. Finally, MD simulations were carried out with NPT conditions and removing position restraints. Coordinates, velocities, and energies were saved every 2 ps. The particle mesh Ewald (PME) algorithm^55^ was used for the long electrostatic interactions and a Verlet scheme for the short range electrostatic and Vander walls interactions with a cut-off for the real space term of 1 nm. The temperature and pressure of the system were maintained constant by the modified Berendsen thermostat and the Parrinello-Rahman barostat, respectively. The constrain of all bond lengths was done using the LINCS algorithm^56^. Runs were performed in a High-Performance-Computing Linux Cluster (HorUS) with a total of 170-cores (4-8 logical cores per node), connected by an HP Procurve 48G–2900 1000T-48 Port switch (Centre of Geosciences, UNAM Campus Juriquilla) and in a High-Performance-Computing Linux Cluster (ADA) with a total of 292 cores connected by a Mellanox Infiniband at 40 Gpbs (National Laboratory of Advance Scientific Visualization, UNAM Campus Juriquilla). All runs were performed in a message passing interface (MPI) environment with a total of 135 cores or 6 cores in Horus or ADA, respectively^48,57^. The GROMACS and VMD 1.9.1^58^ softwares were utilized to analyse trajectories and visualize simulations. The GROMOS96 54A7 (united-atom) and AMBER (all-atom) force fields were also used under the same conditions.

### Analysis of MD simulations

The GROMACS software tools, gmx_gyrate and gmx_sasa, determined the radius of gyration (compression) and the total molecular area, respectively. The GROMACS tools gmx_rmsf and gmx_rms evaluated the RMSF and total RMSD, respectively. RMSF and velocity per residue over time and secondary structure were evaluated using the timeline plugin of VMD 1.9.1^58^. The principal component analysis^59^ used the GROMACS inbuilt tool gmx_covar to yield the eigenvalues and eigenvectors, whereas the gmx_anaeig tool analysed and plotted the eigenvectors. The secondary structure was analysed with the gmx_helix GROMACS tool. The free energy landscape analysis^60^ was obtained by estimating the joint probability distribution from essential plane made by the top two eigenvectors^61^. For the SASA analysis, GROMACS gmx_sasa tool per residue and the POPS^62^ software version 1.8.0, were utilized. The graphs of the trajectory analysis were generated using Xmgrace (Paul J. Turner, Centre for Coastal and Land-Margin Research Oregon Graduate Institute of Science and Technology Beaverton, OR). The automated Poisson-Boltzmann electrostatic calculation was performed using the APBS^63^-PDB2PQR^64^ software. The molecules and trajectories were visualized using VMD 1.9.1^58^ software into the high performance CAVE immersive 3D visualization system VisCube C4 (Visbox, IL). The molecular hydrophobicity potential (MHP) and the solvent accessible surface were projected and scored with PLATINUM software^20^. The protein surface topography (PST) method was performed in combination with the analysis of the Conolly surface as described previously^21^. The inkscape 0.92 software (Inkscape Project. URL https://inkscape.org) was used to generate the graphical artwork. HELANAL^65^ software and Bendix VMD-plugin^66^ were used to analyse the geometry and kink of helices, and the inter-helix angle analysis was performed with the UCSF Chimera package^67^ with the Axes/Planes/Centroids tool. For the evaluation of salt bridges the web server ESBRI^19^ was utilized. For calculation of minimal distance the GROMACS gmx_mindist tool was used. Aggrescan3D^32^ and the Tango^33^ software were used for the prediction of sequence-dependent aggregation properties.

### Construction of lentiviral vectors coding for recombinant vasoinhibins

Vasoinhibin cDNA was generated by PCR from human PRL cDNA including its corresponding signal peptide cloned in the pcDNA3 vector downstream of the CMV promoter, a BamH1 restriction site (5′-G’GATCC-3′), and a Kozak sequence (5′-GCCACC-3′). A pCMV forward primer 5′-CCCACTTGGCAGTACATCA-3′, and reverse primers SalI 5′-TTTTTT*GTCGAC***TTA**GTCTTTTTGATTCATCTGTTGGGC-3′ or SalI 5′-TTTTTT*GTCGAC***TTA**GGTTTGCTCCTCAATCTCTACAGC-3′ were used to construct the cDNAs corresponding to the vasoinhibin of 79 residues or the vasoinhibin of 123 residues, respectively. A stop codon (**TTA**) and a SalI restriction site (*GTCGAC*) were created at the Phe-80 codon for the vasoinhibin of 79 residues or at the Lys-124 codon for the vasoinhibin of 123 residues. For the empty vector, a random non-coding sequence of 20 bp, with sticky ends for BamH1 and SalI was designed, and built-in by pairing the empty vector forward 5′-GATCCTAACGAGCAAAACACGTATAAG-3′ and reverse 5′-TCGACTTATACGTGTTTTGCTCGTTAG-3′ primers. The PCR products and the non-coding fragment were cloned into the pLenti CMV-GFP-Puro (AddGene # 658-5) plasmid conferring puromycin resistance (a gift from Eric Campeau and Paul Kaufman^68^) via enzymatic digestion and ligation using restriction enzymes BamH1 (Jena Biosciences, Jena, Germany) and SalI (New England Biolabs, Ipswich, MA) and T4 DNA ligase (Fermentas, Waltham, MA). The ligated plasmids (pLenti CMV-Vi79-Puro, CMV-Vi123-Puro, and pLenti CMV-non-coding-Puro) were transformed in E. coli competent cells by heat shock and the colony forming units selected with 100 *μ*g ml^−1^ ampicillin (Sigma-Aldrich, St. Louis, MO). The plasmids were grown and purified (Plasmid *Plus* Maxi Kit, QIAGEN, Hilden, Germany) and their correct construction confirmed by restriction analysis and DNA sequencing. The lentiviral particles were produced by co-transfecting the human embryonic kidney cell line (HEK293 T/17 obtained from ATCC Manassas, VA) with the pLenti CMV-Vi79-Puro, CMV-Vi123-Puro or the CMV-non-coding-Puro together with the packaging plasmids: pMDLg/pRRE (Addgene # 12251), pRSV-Rev (Addgene # 12253), and pMD2.G (Addgene # 12259) (gifts from Didier Trono^69^). Conditioned medium containing lentiviral vectors was harvested every 12 h for 2 days, pooled, and titrated through a puromycin kill curve in 293T/17 cells.

### Recombinant vasoinhibin production by stably transduced HEK293T/17 cells

HEK293T/17 cells were cultured in Dulbecco’s modified eagle medium (DMEM) supplemented with 10% of foetal bovine serum (FBS) and antibiotics (100 U ml^−1^ of penicillin-streptomycin/100 *μ*g ml^−1^ normosin). HEK293T/17 cells were stably transduced with recombinant lentiviral vectors coding for the vasoinhibins of 79 and 123 residues or with the empty vector (pLenti-CMV-non-coding-Puro), selected with 3 *μ*g ml^−1^ puromycin (Sigma) for 24 h and expanded. Conditioned medium containing vasoinhibins was harvested every 48 h for 2 weeks, pooled, and stored at −70 °C.

### Tricine SDS-PAGE Western Blot

Tricine SDS-PAGE on a 16% acrylamide gel, followed by immunoblotting with polyclonal anti-human PRL antiserum (CL-1)^70^ or monoclonal anti PRL N-terminal antibody 5602^15^ were used for evaluation and quantitation, respectively, the presence of vasoinhibins in the conditioned medium of transduced cells. Tricine-SDS-PAGE is the preferred electrophoretic system for the resolution of proteins smaller than 30 kDa^71^. Detection was by chemiluminescence using the peroxidase affiniPure donkey anti-rabbit or anti-mouse IgG (H&L) (Jackson ImmunoResearch, West Grove, PA) and the SuperSignal West Pico PLUS Substrate kit and the Protein simple fluorchem imager and gel documenter system (both from ThermoFisher Scientific, Waltham MA). Following the method described previously^37,72^, the vasoinhibin concentration was quantified by interpolating from a PRL standard curve (run within the same blot) the combined densitometric values of the two vasoinhibin isoforms in each conditioned media (Supplementary Fig. 7e,f). The removal of N-linked oligosaccharides with Peptide N-glycosidase F (New England Biolabs) was done according to manufacturer instructions to evaluate the presence of glycosylated vasoinhibins in the conditioned media of the transduced cells. The Quantity One 1-D Analysis Software (BioRad) evaluated optical density values.

### Endothelial cell proliferation

Bovine pulmonary artery endothelial cells (CPAE) acquired from the ATCC (Manassas, VA) and bovine umbilical vein endothelial cells (BUVEC) obtained as described^73^ were maintained in Eagle’s minimum essential medium (EMEM) supplemented with 20% of FBS and in F12K medium with 10% FBS, respectively, supplemented with antibiotics (100 U ml^−1^ of penicillin-streptomycin/100 *μ*g ml^−1^ normosin). To test the proliferative effect of the recombinant vasoinhibins of 79 and 123 residues, CPAE or BUVEC were seeded at 6000 cells cm^−2^ in a 96-well plate, starved with 0.5% FBS for 24 h, and treated with basic fibroblast growth factor (10 ng ml^−1^) in the absence of presence of 50 *μ*l (for CPAE) or different volumes (for BUVEC) of the conditioned medium of HEK293T/17 cells transduced with empty lentiviral vectors, lentiviral vectors coding vasoinhibins of 79 or 123 residues, or of non-transduced cells. CPAE or BUVEC were allowed to proliferate for 24 h and the number of cells with newly synthesized DNA were evaluated by the click reaction^74^. Briefly, cells were treated with 10 *μ*M of the thymidine analogue 5-ethynyl-2′-deoxyuridine (EdU) (Sigma-Aldrich) during the 24 h treatment with the different conditioned media. Cells were then fixed with paraformaldehyde 4% (20 min) and permeabilized with 0.5% TritonX-100 in 1X TBS (1 h). Between each step, cells were washed with 1X TBS for 2 min in an orbital shaker. The EdU present in the nuclei was detected by fluorescent-azide labelling in a 20 min click reaction performed using a fresh mix of 100 mM Tris, 0.5 mM CuSO_4_, 50 mM ascorbic acid, and 20 *μ*M Azide Fluor 545 (Sigma-Aldrich); followed by a 2 and a 30 minutes wash step with 0.5% TritonX-100 in 1X TBS. Nuclear DNA was counterstained with 5 *μ*g ml^−1^ bisBenzimide Hoechst 33342 trihydrochloride (Sigma-Aldrich) as reported^75^. Images were digitalized in a fluorescence inverted microscope (Olympus IX51, Japan), analysed using the CellProfiler software^76^, and merged with ImageJ 1.X software^77^. In other experiments DNA synthesis was quantified by [^3^H]-thymidine incorporation. Briefly, CPAE were seeded at a density of 500 cells cm^−2^ in a 48 well-plate, cultured with 0.5% of FBS for 24 h together with 20 *μ*l of the different conditioned media with or without vasoinhibins. During the treatment, cells were pulsed with 0.5 *μ*Ci ml^−1^ of [^3^H]-thymidine (Perkin Elmer, Boston, MA) and the measurement of [^3^H]-thymidine incorporation was performed as described^37^.

### Cell Migration Assay

Cell Migration was assessed by the scratch wound healing assay^24^. CPAE were seeded at confluence in a 6-well plate in medium containing 0.5% serum for 24 h and scratched using the edge of a cell scraper. The medium was then replaced by one with 10% of serum and diluted 50% with conditioned medium of HEK293T/17 cells transduced with the empty lentiviral vector, lentiviral vectors coding vasoinhibins of 79 and 123 residues, or of non-transduced cells. After 24 h, migration was recorded using an Olympus IX51 microscope (Olympus instrument) and analysed with the CellProfiler^76^ software and the wound healing pipeline.

### Proliferation of endothelial cells transduced with recombinant lentiviral vectors coding for the vasoinhibins of 79 and 123 residues

BUVEC cells seeded in a 6 well plate at a density of 10,000 cells cm^−2^ were stably transduced with lentiviral vectors coding for the vasoinhibins of 79 and 123 residues or with the empty vector in the presence of 5 *μ*g ml^−1^ polybrene (Sigma-Aldrich). Culture medium was changed at 24 h post-transduction and cells were counted every 24 hours for 3 days. At 72 h post-transduction nuclear DNA was stained with 2.5 *μ*g ml^−1^ bisBenzimide Hoehcst 33342 trihydrochloride. Photographs were obtained in an Inverted Basic Microscope IX51 (Olympus, Tokyo, JP), with reflected fluorescence system dichroic mirror DM400. Nuclei number was automatically determined using Image J software.

### Quantitative real-time PCR (qRT-PCR)

CPAE cells were seeded in a 12-well plate until reaching 90% confluency. Cells were then placed in 0.5% of FBS for 24 h followed by changing the medium to one diluted 50% with conditioned media of HEK293T/17 cells transduced with empty lentiviral vectors, lentiviral vectors coding vasoinhibins of 79 and 123 residues, or of non-transduced cells. After 4 h total RNA was isolated using Trizol reagent (Invitrogen, Carlsbad, CA) and reverse transcribed using the High-Capacity cDNA Reverse Transcription Kit (Applied Biosystems, Foster City, CA, USA). PCR products were detected and quantified using Maxima SYBR Green qPCR Master Mix (Thermo Fisher Scientific) in a final reaction of 10 *μ*l containing template and 0.25 *μ*M of each primer. Amplification was performed in the CFX96 real-time PCR detection system (Bio-Rad) and included a 10 min denaturation step at 95 °C, followed by 35 cycles of amplification (10 sec at 95 °C, 30 sec at the primer pair-specific annealing temperature, and 30 sec at 72 °C). The following bovine primers were used: IL1A forward (5′-TCAAGGAGAATGTGGTGATG-3′) and reverse (5′-CTGGAAGCTGTAATGTGCTG-3′); ICAM1 forward (5′-CGTTAAGCTACACCCACCTT-3′) and reverse (5′-AGGTAAGGGTCTCCATCACA-3′). The PCR data were analysed by the 2^−*ΔΔCT*^ method, and cycle thresholds (CT) normalized to the housekeeping gene cyclophilin A (PPIA) calculated the mRNA levels. PPIA forward (5′-GGTTCCCAGTTTTTCATTTG-3′) and reverse (5′- ATGGTGATCTTCTTGCTGGT-3′) primers were used.

### Statistical analysis

The GraphPad Prism 5.0 software (GraphPad Software, La Jolla, CA) was used. One-way ANOVA followed by Bonferroni’s correction test compared differences between more than three groups. The significance threshold was set at *p* < 0.05.

## Electronic supplementary material

Supplementary Information

## Data Availability

All data generated or analysed during this study are included in this published article (and its Supplementary Information file).
