# Supplementary material for: Vasoinhibin comprises a three-helix bundle and its antiangiogenic domain is located within the first 79 residues
Source: Sci Rep. 2018 Nov 20;8:17111. doi: 10.1038/s41598-018-35383-7 (PMC6244167; doi:10.1038/s41598-018-35383-7)
Supplement: Supplementary file 1 — Supplementary Information [file 41598_2018_35383_MOESM1_ESM.pdf]

Supplementary Information

Vasoinhibin comprises a three-helix bundle and its antiangiogenic domain is located within the first 79 residues

Juan Pablo Robles[1], Magdalena Zamora[1], José Luis Velasco-Bolom[2], Miriam Tovar[1], Ramón Garduño-Juarez[2], Thomas Bertsch[3], Gonzalo Martínez de la Escalera[1], Jakob Triebel[3], and Carmen Clapp[1,*]

[1]Instituto de Neurobiología, Universidad Nacional Autónoma de México (UNAM), Querétaro, México.

[2]Biofísica y Ciencia de Materiales, Instituto de Ciencias Físicas, UNAM, Cuernavaca, México

[3]Institute for Clinical Chemistry, Laboratory Medicine and Transfusion Medicine, Nuremberg General Hospital & Paracelsus Medical University, Nuremberg, Germany;

***Correspondence should be addressed to C.C. (clapp@unam.mx)**

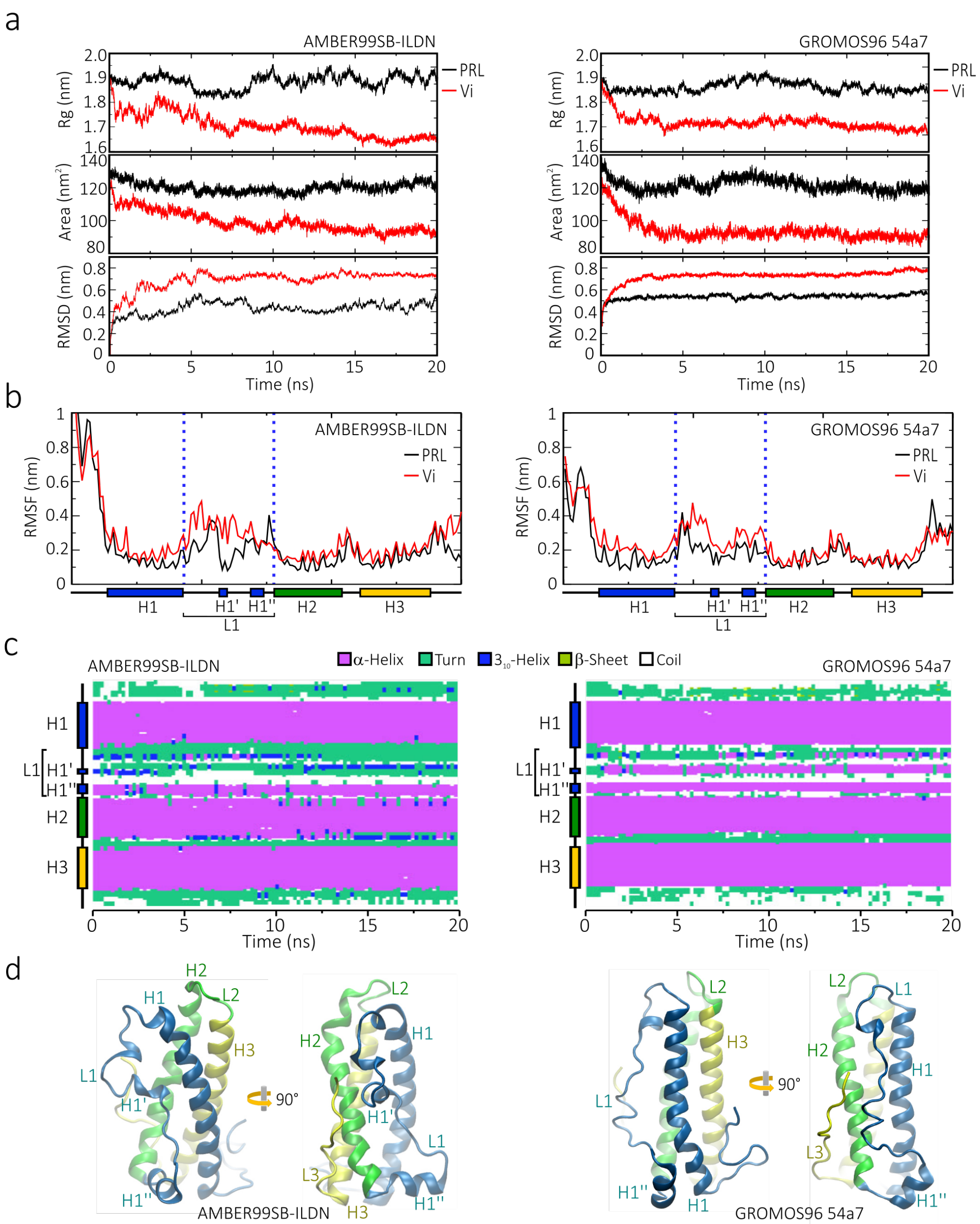

Supplementary Figure 1. Trajectory analysis and secondary structure of PRL and a vasoinhibin of 150 amino acids (Vi) with AMBER99SB-ILDN and GROMOS96 54a7 force fields. a. Radius of gyration (Rg), surface area, and root mean square deviation (RMSD) during 20 ns of molecular dynamic simulation (MD). **b**. Total root mean square fluctuation (RMSF) per residue of Vi relative to PRL. Loop 1 (L1) is flanked by blue pointed lines. **c.** Secondary structure along the residue sequence of Vi. **d.** Representative snapshot of vasoinhibin structure in a front and a lateral view.

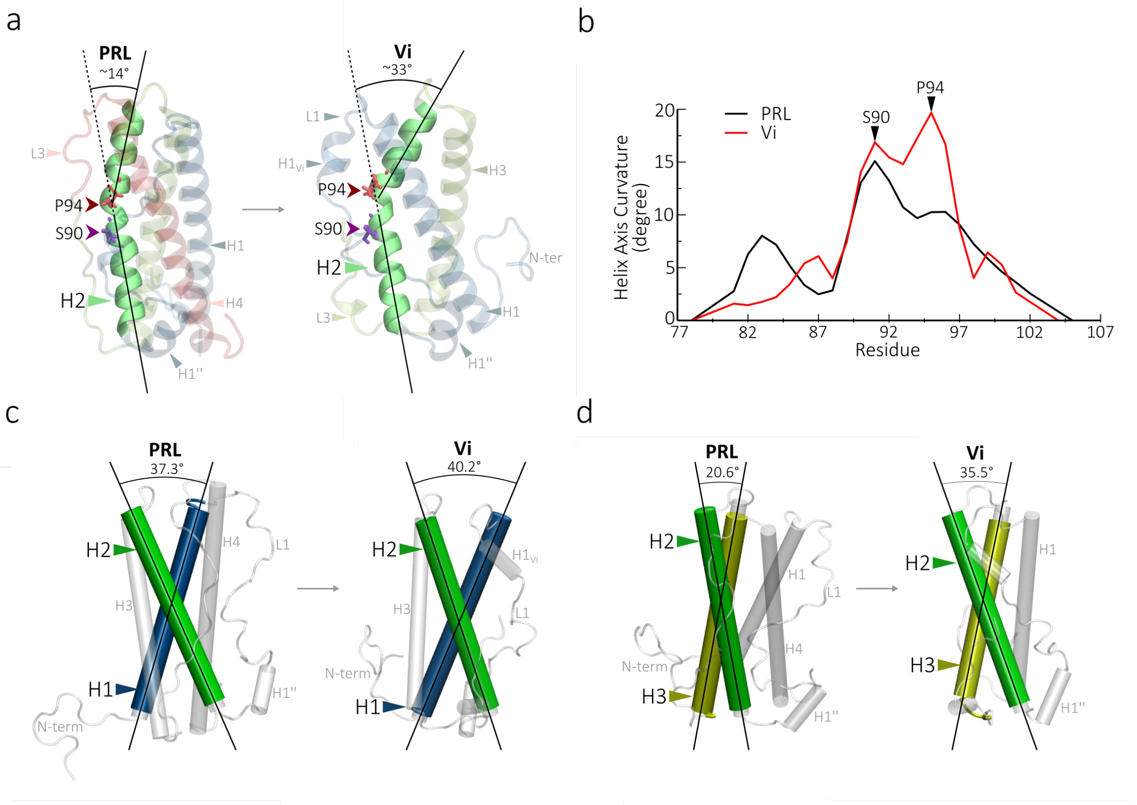

Supplementary Figure 2. Analysis of α-helices geometry and crossing angles in PRL and a vasoinhibin of 150 amino acids (Vi). a. Cartoon representation showing the α-helix 2 (H2) kink and bending angles in PRL and Vi. The residues P94 and S90 are indicated in each molecule (arrows). **b.** Quantitation of the curvature along the H2 axis in PRL and Vi. Maximal bending points occur at S90 and P94 in PRL and Vi, respectively. Crossing angle analysis between H1-H2 **(c)** and H2-H3 **(d)** in PRL and Vi.

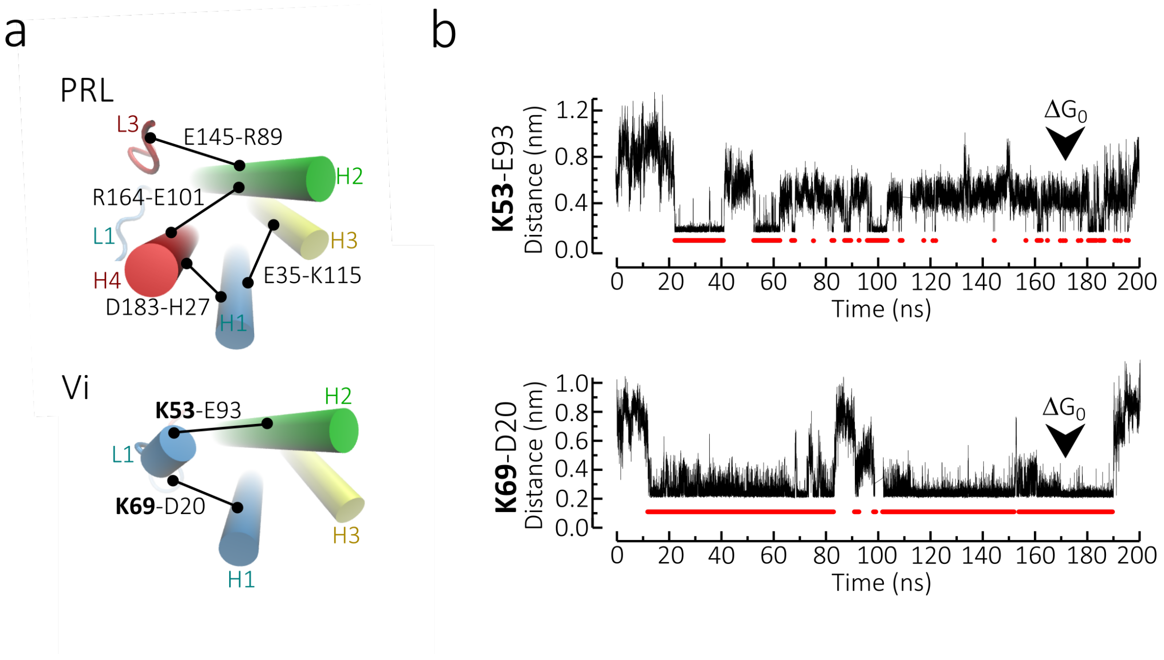

Supplementary Figure 3. Analysis of salt-bridge pairs formed by buried basic residues K53 and K69 in a 150 residue vasoinhibin (Vi). a. Schematic representation of α-helices (H1-4) and loops (L1, L3) in PRL (pdb 1RW5) and Vi (frame 175872) visualized from the top to illustrate the inter-helical salt-bridges. No salt-bridge is found between L1 and other part of the molecule in PRL, where most salt-bridges occur within lateral structures, except for R164-E101 present in the core of the molecule. Residues K53 and K69, buried in Vi, are located in L1 and form salt-bridges with E93 in H2 and N20 in H1, respectively. **b.** Analysis of the minimum distance between the residues in salt-bridges K53-E93 and K69-D20 during 200 ns of molecular dynamic simulation. The red line indicates the minimum distance reached by the residue pairs qualifying as forming a salt-bridge. Head-arrow indicates the frame with the lowest Vi energy structure (ΔG0).

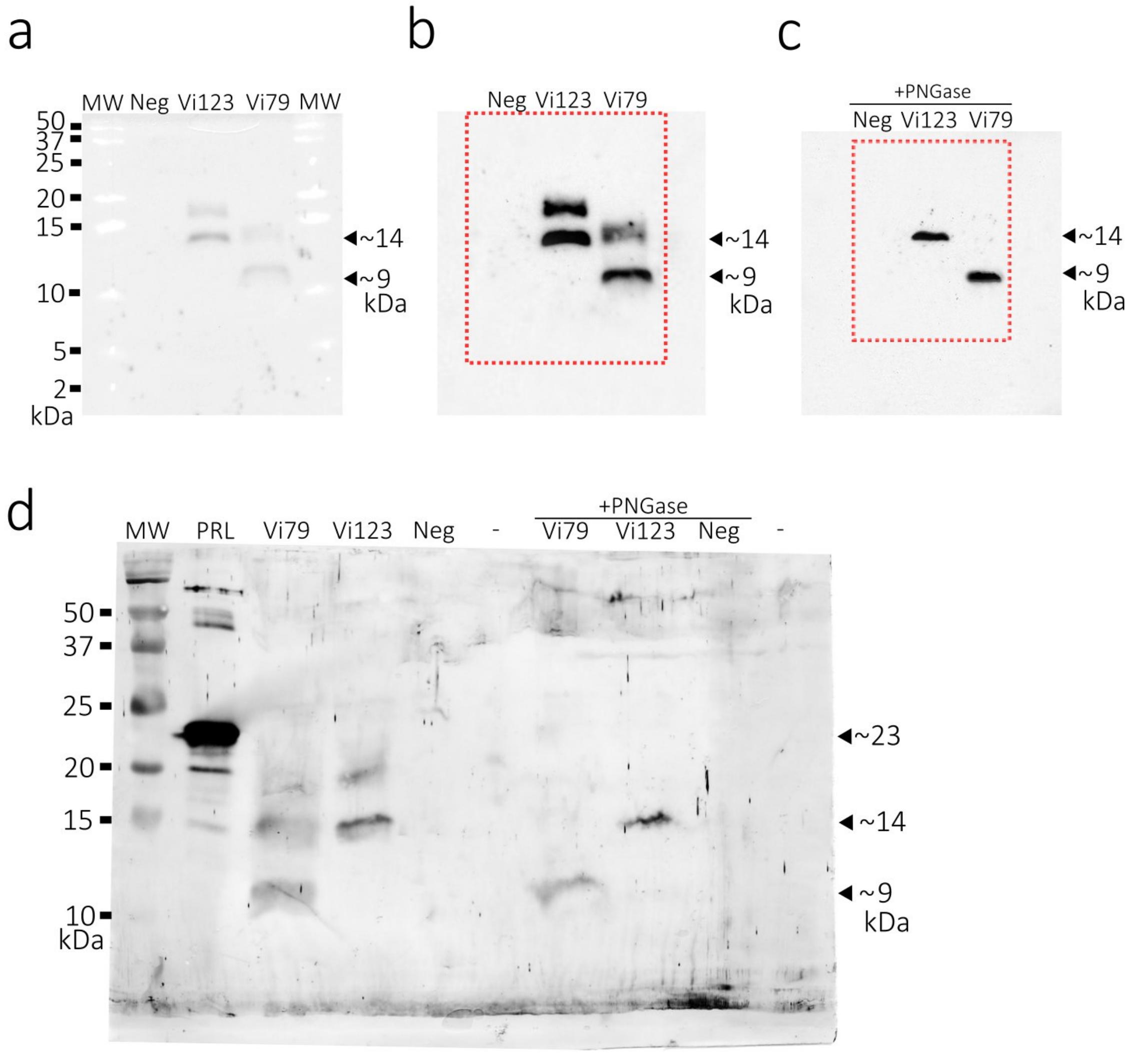

Supplementary Figure 4. Analysis of the production of recombinant vasoinhibin comprising 1 to 79 amino acids (Vi79) of PRL a. Full unedited Western blot of conditioned media (CM) obtained from HEK293T/17 cells stably transduced with lentiviral vectors encoding the first 79 residues (calculated molecular weight ~9 kDa) or the first 123 residues (~14 kDa). CM of cells transduced with empty lentiviral vector as negative control (Neg), molecular weight marker (MW) bands are superimposed automatically. **b.** and **c.** Full unedited gel for figure 7a and b, respectively, pointed line red rectangles correspond to cropped images within the manuscript. **d.** Full unedited Western blot analysis of conditioned media (CM) of figure 7 a and b, without or with PNGase treatment, developed with alkaline phosphatase method. Full length PRL was used as positive control.

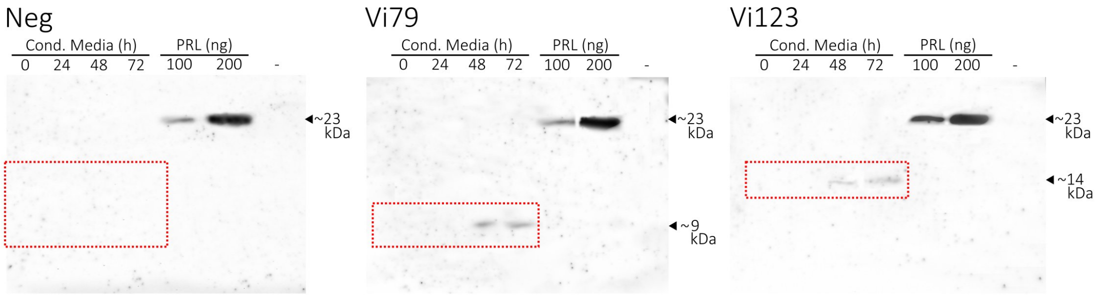

Supplementary Figure 5. Unedited blots of figure 9d. Full unedited gels from figure 9d pointed line red rectangles correspond to cropped images within the manuscript.

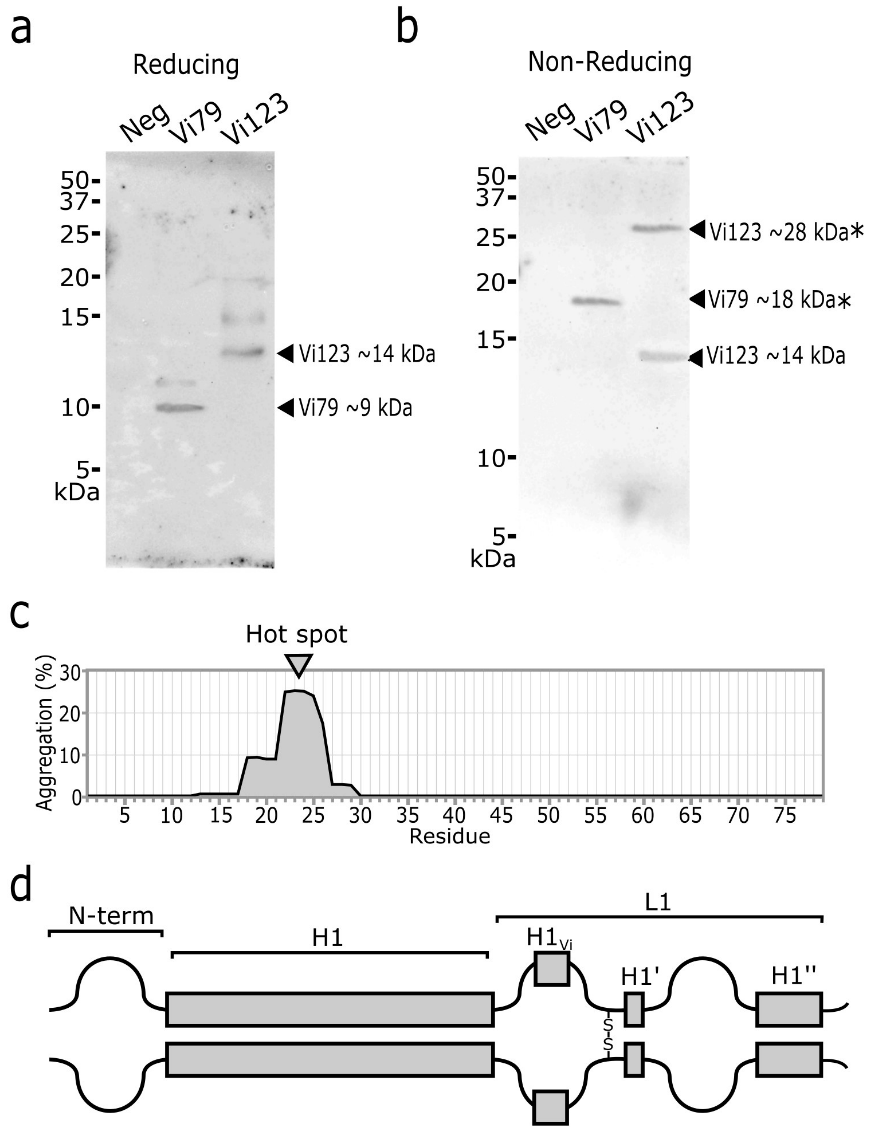

Supplementary Figure 6. Analysis of the disulphide bond dimerization of 79- and 123-residue vasoinhibins. Reducing **(a)** and non-reducing **(b)** Tricine SDS-PAGE Western blot analysis of conditioned media (CM) from HEK293T/17 cells stably transduced with lentiviral vectors encoding the first 79 residues (Vi79) or the first 123 residues (Vi123) of human PRL. CM of cells transduced with empty lentiviral vector was the negative control (Neg). Monomers of Vi79 and Vi123 have ~9 kDa and ~14 kDa, respectively. Dimers of Vi79 (~18 kDa) and Vi123 (~28 kDa) are indicated (*). Numbers on left indicate the molecular weight of marker proteins. **c.** Aggregation per residue of Vi79 as predicted by the Aggrescan3D and Tango plot sofware analyses. The major aggregation hot spot is indicated (arrow). **d.** Proposed model for the Vi79 dimer conformation. Disulfide bridge C58-C58 is indicated (-S-S-).

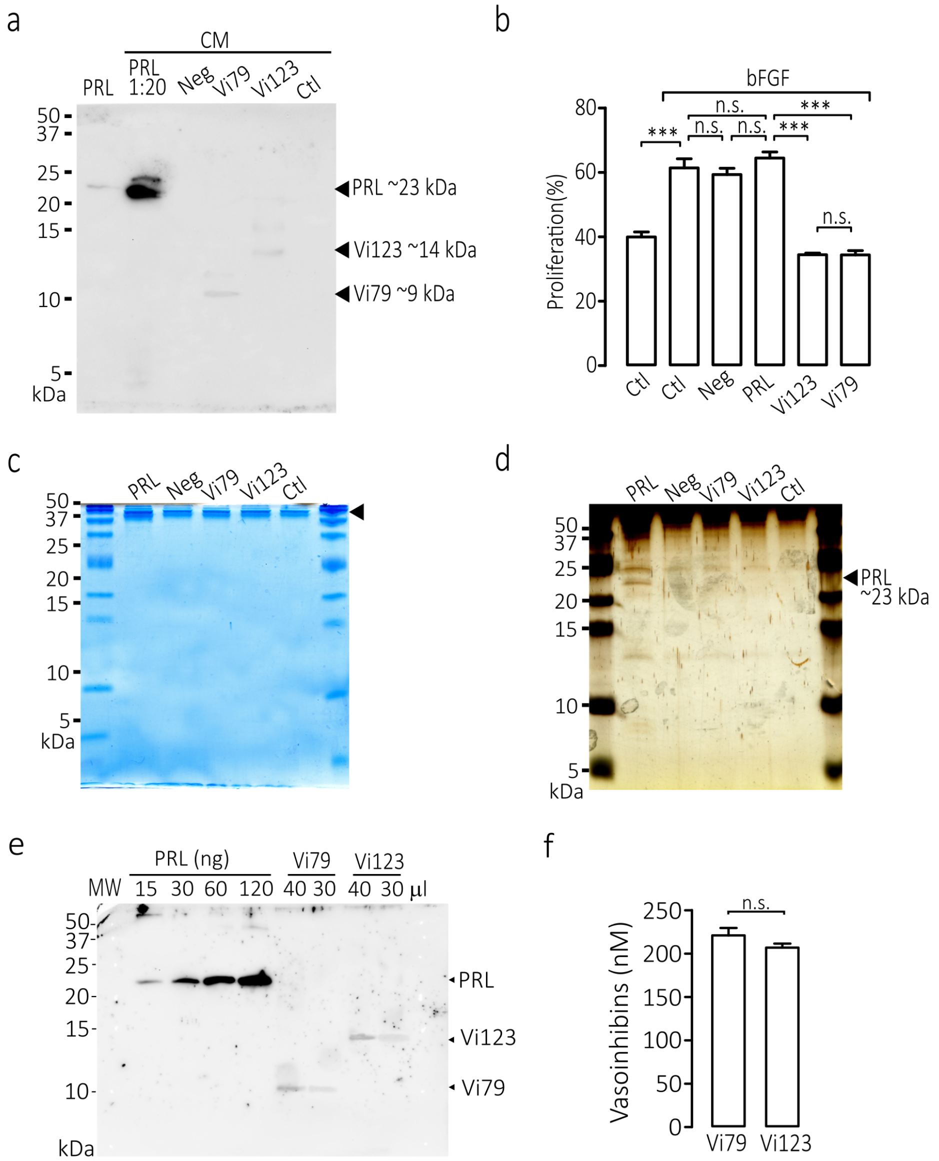

Supplementary Figure 7. Bioactivity of PRL and PRL fragments of 79 and 123 residues. a. Western blot analysis of the conditioned media (CM) obtained from HEK293T/17 cells stably transduced with lentiviral vectors encoding full-length PRL, the first 79 residues of PRL (Vi79), the first 123 residues of PRL (Vi123) or with an empty vector (Neg). The PRL CM was diluted 20 times. A PRL standard (PRL, 20 ng) was run as reference. Numbers on left indicate the molecular weight of marker proteins. **b.** Proliferation of bovine pulmonary artery endothelial cells (CPAE) quantified by the Edu-click reaction and expressed relative to the total number of cells in the field. Cells were incubated for 24 h with or without basic fibroblast growth factor (bFGF) in combination or not with the same volume of CM without (Neg) or with full-length PRL (undiluted CM), Vi79 or Vi123. *** $p<0.001$. n.s., non-significant. Total protein level in the different CM assessed by Coomassie blue **(c)** and silver stain **(d)**. **e.** Western blot of a PRL standard curve and two different volumes of CM containing Vi79 or Vi123. **f.** The concentration of Vi79 and Vi123 in CM were obtained by interpolating from the PRL standard curve the desitometric values of the vasoinhibin isoforms in each CM. Values are means ± S.E.M. from 3 independent determinations.

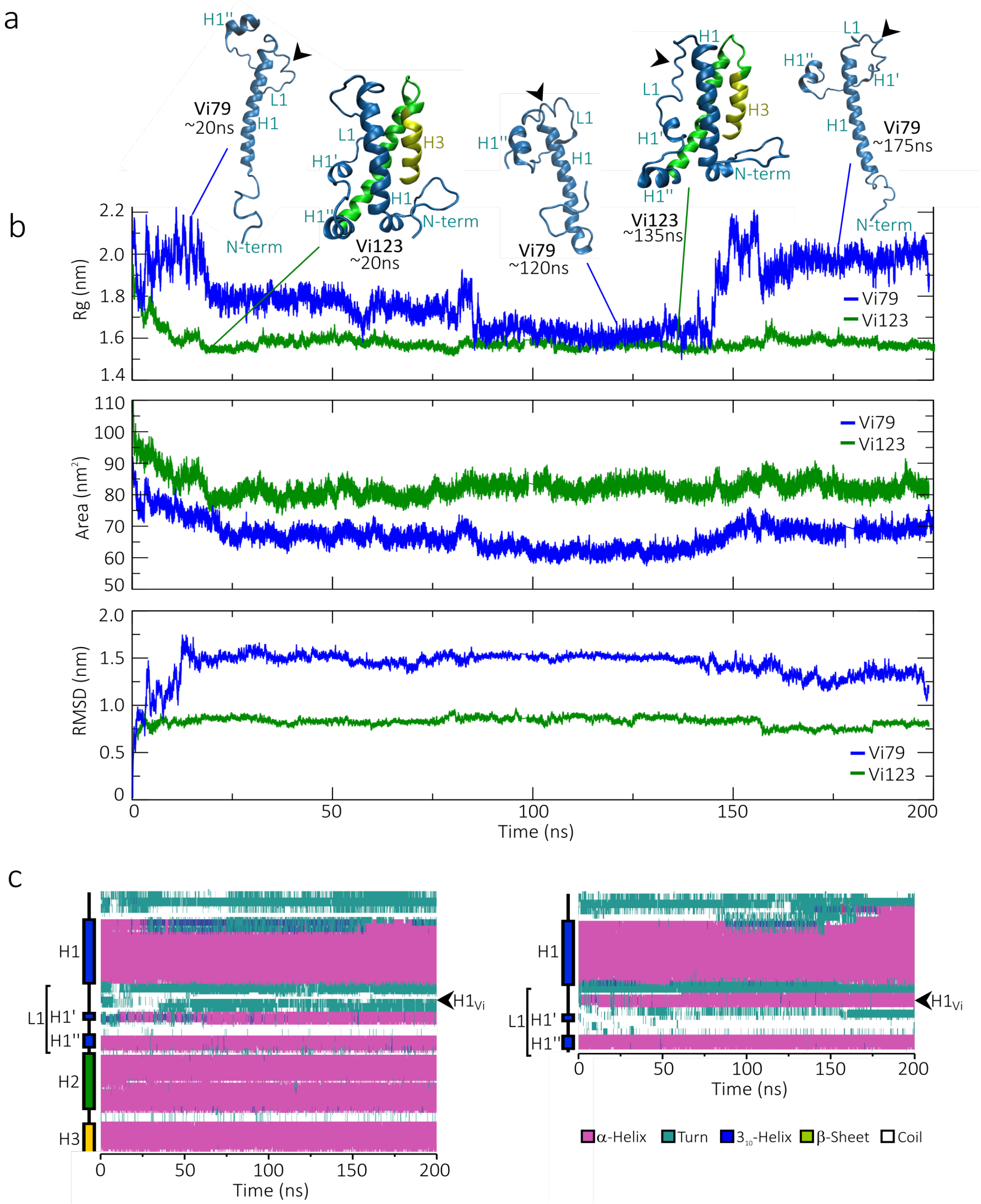

Supplementary Figure 8. Analysis of the 123- and 79-residue vasoinhibins (Vi) during 200 ns of molecular dynamic simulation (MD). a. Representative snapshots of Vi123 and Vi79 at the indicated times (lines) throughout the MD. The $H1_{Vi}$ region in each structure is indicated (arrow). **b.** Radius of gyration (Rg), surface area, and root mean square deviation (RMSD). **c.** Changes in the secondary structure along the residue sequence of Vi123 (left) and 79 (right) during the 200 ns MD. The colours represent different secondary structures according to the code.
